# Prognostic value of an autophagy-related long-noncoding-RNA signature for endometrial cancer

**DOI:** 10.18632/aging.202431

**Published:** 2021-02-01

**Authors:** Xiufang Wang, Chenyang Dai, Minqing Ye, Jingyun Wang, Weizhao Lin, Ruiman Li

**Affiliations:** 1 Department of Obstetrics and Gynecology, The First Affiliated Hospital of Jinan University, Guangzhou 510632, China; 2 Department of Obstetrics and Gynecology, Foshan Women and Children Hospital, Foshan 528000, China

**Keywords:** endometrial cancer, autophagy, long-noncoding-RNA, TCGA, prognostic signature

## Abstract

This study retrieved the transcriptome profiling data of 552 endometrial cancer (EC) patients from the TCGA (The Cancer Genome Atlas) database, and identified 1297 lncRNAs (long noncoding RNAs) related to autophagy genes using Pearson correlation analysis. Univariate Cox regression analysis of the training data set revealed that 14 autophagy-related lncRNAs had significantly prognostic value for endometrial cancer (*P* < 0.01). Multivariate Cox regression analysis of these autophagy-related lncRNAs established the following autophagy-related lncRNA prognosis signature for endometrial cancer: PI = (0.255 × AC005229.4 expression) + (0.405 × BX322234.1 expression) + (0.169 × FIRRE expression value) + (–0.122 × RAB11B-AS1 expression) + (–0.338 × AC003102.1 expression). This signature was validated in both the testing data set and the entire data set. The areas under the receiver operating characteristics curves for the 1-, 3-, and 5-year overall survival rates in the entire data set were 0.772, 0.733, and 0.714, respectively. In addition, a gene set enrichment analysis confirmed that cancer-related and autophagy-related pathways were significantly up-regulated in the high-risk group. In summary, this study has demonstrated that a signature comprising five autophagy-related lncRNAs has potential as an independent prognostic indicator of endometrial cancer, and also that these lncRNAs may play a key role in the development of endometrial cancer.

## INTRODUCTION

Endometrial cancer (EC) is a common malignant tumor in gynecology that seriously threatens the physical and mental health of females. The latest data from the American Cancer Society indicate that EC is the most-common malignant tumor of the female reproductive system in the United States [1]. EC has also become the second-most-common gynecological malignant tumor (after cervical cancer) in China, where its incidence is increasing and the mean onset age is decreasing. Although surgery is effective for treating most patients with early-stage EC, the prognosis of cases at stages III and IV remains very poor, with 5-year overall survival (OS) rates of 47~69% and 15~17%, respectively [2], and there remains a risk of recurrence or metastasis even after surgery in some EC patients. This situation indicates the importance of the early identification of EC patients at high risk of recurrence and metastasis.

The indicators that are commonly used in clinical risk assessments of EC are mainly based on clinicopathological characteristics such as the pathological tissue type, tumor pathological grade, FIGO stage, muscle invasion depth, and tumor size [3]. Advanced age and lymphatic vascular space invasion are also predictors of a poor prognosis in patients with EC [4, 5]. However, these prognosis criteria and classifications of EC have limitations in clinical practice that make them unsuitable for accurately predicting the prognosis of EC patients [6, 7]. This may lead to inaccurate assessments of the condition of EC patients, and hence either undertreatment or overtreatment. There is therefore an urgent need for effective indicators of the prognosis to be identified in order to help EC patients with more-targeted treatment options so as to improve their prognosis. In short, the development of new predictive biomarkers is essential for the pathogenesis, prognosis, evaluation, and biological treatment of EC.

Autophagy is a degradation pathway that is highly conserved during the evolution of eukaryotes. The formation of a double-layer membrane structure allows the transportation of damaged organelles, misfolded and aggregated proteins, and other macromolecular substances to the lysosome for degradation or recycling [8]. Autophagy plays very complex roles in tumors, including inhibiting or promoting them in different environments and stages of cancer development [9, 10]. Autophagy is generally beneficial during the normal state of the body and the early stages of tumors, by eliminating oncogenic protein substrates, misfolded proteins, and damaged organelles, maintaining cell homeostasis, and either preventing tumors from occurring or inhibiting their progression [11]. However, once tumor develop to an advanced stage, autophagy—as a dynamic degradation and recycling system—promotes their survival and growth by enhancing the living ability of cancer cells in an environment characterized by nutrient starvation and hypoxia [12, 13]. Autophagy can also enhance the resistance of tumors to anticancer treatments such as radiotherapy, chemotherapy, and targeted therapy [14].

The dynamic role of autophagy in tumor progression has received considerable attention in research into clinical treatments. Regulating autophagy activity to inhibit tumor development has emerged as a new direction for tumor treatments. Autophagy and EC are closely related, with studies showing that autophagy plays a vital role in the development and survival mechanism of EC [15]. Giatromanolaki et al. and Deng et al. found that certain autophagy-related factors are overexpressed in EC tissues and can promote the occurrence and development of these tumors [16, 17]. The PI3K-Akt-mTOR signal transduction pathway is often overactivated in EC [18], and autophagy inhibitors such as rapamycin and chloroquine can inhibit the proliferation of EC cells [19, 20]. Autophagy is therefore a potential target for exploring the pathogenesis of EC.

Long noncoding RNA (lncRNA) is a noncoding RNA longer than 200 nucleotides that has no protein coding function. This type of RNA can participate in regulation via various mechanism, such as epigenetic regulation, transcription regulation, and posttranscriptional regulation. Gene expression plays an important role in various biological processes such as cell proliferation, differentiation, and apoptosis [21–23]. lncRNAs have been shown to be closely related to human diseases, especially those involving tumors [24, 25]. lncRNAs are abnormally expressed in a broad spectrum of tumors, and they play a key role in tumor occurrence, metastasis, and chemotherapy resistance, including in EC [26, 27]. lncRNAs the proliferation, migration, and invasion of EC cells by participating in various signal pathways, and they are potential targets for EC therapy and biomarkers for early diagnoses [28].

Autophagy is an important regulatory pathway for tumors that is closely related to lncRNA. Autophagy and lncRNA work together in tumors and other human diseases [29]. Many lncRNAs are involved in the dynamic process of autophagy, and can regulate the progression of most tumors by regulating the transcription and posttranscriptional autophagy-related genes [30, 31]. Example of this include AC023115.3 lncRNA, which increases the chemosensitivity of glioma cells to cisplatin by inhibiting autophagy [32]. Conversely, Li et al. found that MALAT1 lncRNA promotes the progression of pancreatic cancer by enhancing autophagy [33], while AC023115.3 lncRNA improves the chemosensitivity of glioma cells to cisplatin by regulating the miR-26a-GSK3β-Mcl1 pathway. Long-chain noncoding MEG3 interacts with ATG3 so as to increase the level of autophagy, resulting in inhibition of the occurrence and development of epithelial ovarian cancer [34]. LncRNAs, specifically HOTAIR, contribute to the cisplatin resistance of EC cells by enhancing autophagy [35]. Since these autophagy-related lncRNAs play important regulatory roles in the proliferation, metastasis, and chemotherapy resistance of tumor cells, they may be useful for prognosis evaluations of EC patients and as potential therapeutic targets for EC.

This study analyzed the lncRNAs data of EC patients in the TCGA (The Cancer Genome Atlas) database, identified autophagy-related lncRNAs related to the prognosis of EC, and constructed a novel autophagy-related lncRNA prognosis signature for EC. The present findings provide new ideas and directions for future investigations of the pathogenesis and prognosis of EC.

## RESULTS

### Identification of autophagy-related lncRNAs in EC

We extracted 14,142 lncRNA data sets and 210 autophagy-related genes from the TCGA database. The coefficients for the correlations between lncRNAs and autophagy-related genes were calculated using Pearson correlation. Applying screening criteria of a correlation coefficient of >0.3 and *P*<0.001 resulted in the identification of 1297 autophagy-related lncRNAs.

### Construction of a signature of five autophagy-related lncRNAs for patients with EC

**Figure 1:**
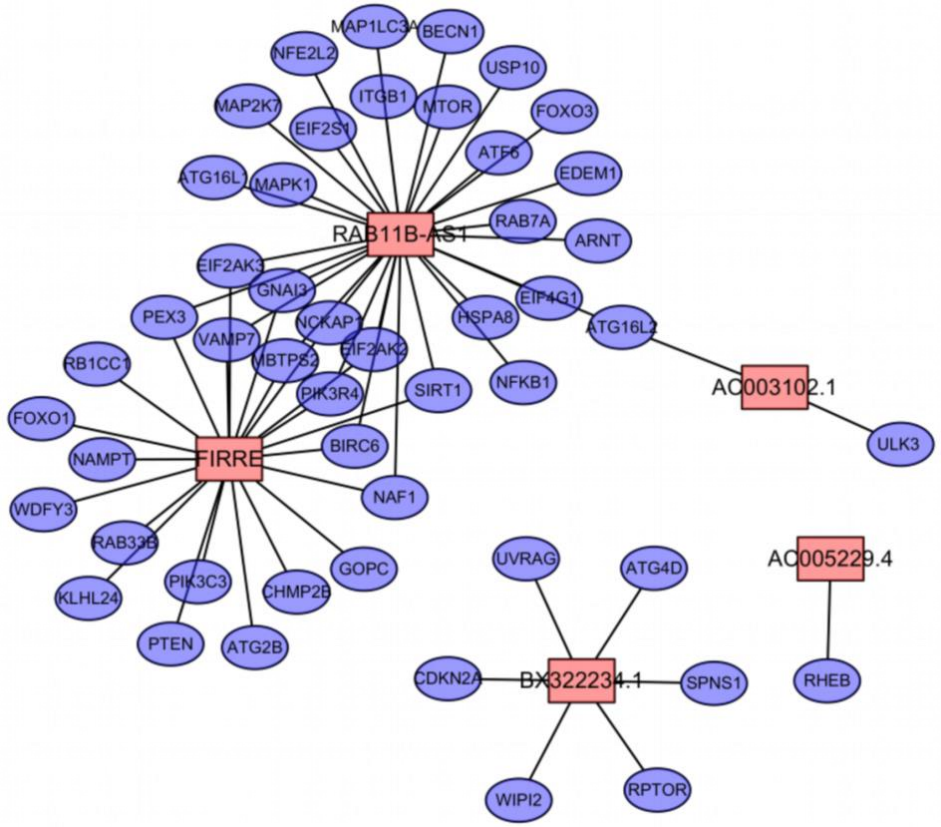
**The co-expression network of OS-associated lncRNAs and autophagy genes in endometrial cancer.** Among them, the pink node represents the lncRNA, and the blue node represents the co-expressed autophagy gene.

We used the caret package in R software to randomly divide the EC samples into the training and testing data sets. Applying univariate Cox regression analysis to the training data set revealed 14 autophagy-related lncRNAs that had a significant prognostic value for EC (*P*<0.01). The detailed information of 14 autophagy-related lncRNA significantly related to OS are presented in Table 1. The following autophagy-related lncRNA prognosis signature was established for EC: PI = (0.255 × AC005229.4 expression) + (0.405 × BX322234.1 expression) + (0.169 × FIRRE expression) + (–0.122 × RAB11B-AS1 expression) + (–0.338 × AC003102.1 expression). The positive coefficients for AC005229.4, BX322234.1, and FIRRE in this signature indicate that patients with high expression levels of these lncRNAs had worse survival, whereas those with high expression levels of RAB11B-AS1 and AC003102.1 had better survival. The correlations between these five lncRNAs and autophagy genes are presented in Table 2 and Figure 1.

**Table 1:** Detailed information of 14 autophagy-related lncRNA significantly related to OS in EC.

**lncRNA**	**KM**	**B**	**SE**	**HR**	**HR.95L**	**HR.95H**	***P*-value**
LINC00662	0.002	0.266	0.074	1.305	1.129	1.508	0.000
AC017074.1	0.001	0.047	0.018	1.049	1.012	1.086	0.008
AC079807.1	0.008	0.805	0.204	2.236	1.499	3.334	0.000
LNCTAM34A	0.001	-0.318	0.123	0.727	0.571	0.926	0.010
AC107057.1	0.000	0.096	0.033	1.101	1.033	1.174	0.003
AC003102.1	0.006	-0.418	0.148	0.658	0.493	0.879	0.005
RAB11B-AS1	0.010	-0.204	0.076	0.815	0.703	0.945	0.007
AC005229.4	0.001	0.274	0.088	1.316	1.107	1.564	0.002
KRT7-AS	0.004	0.170	0.052	1.185	1.071	1.312	0.001
BX322234.1	0.002	0.578	0.140	1.783	1.356	2.345	0.000
AC006329.1	0.004	0.106	0.034	1.112	1.040	1.189	0.002
LINC01224	0.005	0.142	0.046	1.153	1.054	1.261	0.002
FIRRE	0.005	0.226	0.063	1.254	1.108	1.419	0.000
AC010894.2	0.003	0.275	0.091	1.317	1.101	1.575	0.003

**Table 2:** Expression correlations between autophagy genes and OS-associated lncRNAs in EC.

**LncRNA**	**ARG gene**	**Correlation**	***P-*value**
AC005229.4	RHEB	0.365765623	6.46E-19
BX322234.1	WIPI2	0.312898199	5.29E-14
BX322234.1	UVRAG	0.446190163	2.32E-28
BX322234.1	SPNS1	0.402240936	6.98E-23
BX322234.1	RPTOR	0.369415813	2.73E-19
BX322234.1	CDKN2A	0.327356943	2.97E-15
BX322234.1	ATG4D	0.313754433	4.48E-14
FIRRE	WDFY3	0.499216145	3.96E-36
FIRRE	VAMP7	0.330521098	1.55E-15
FIRRE	SIRT1	0.328338857	2.43E-15
FIRRE	RB1CC1	0.536857049	1.57E-42
FIRRE	RAB33B	0.383946076	7.87E-21
FIRRE	PTEN	0.401258287	9.07E-23
FIRRE	PIK3R4	0.511169817	4.47E-38
FIRRE	PIK3C3	0.389171215	2.10E-21
FIRRE	PEX3	0.340283484	1.98E-16
FIRRE	NCKAP1	0.51556263	8.23E-39
FIRRE	NAMPT	0.329353888	1.97E-15
FIRRE	NAF1	0.305954124	2.00E-13
FIRRE	MBTPS2	0.405194041	3.17E-23
FIRRE	KLHL24	0.635974705	6.71E-64
FIRRE	GOPC	0.35438881	8.88E-18
FIRRE	GNAI3	0.427794497	5.71E-26
FIRRE	FOXO1	0.318055813	1.93E-14
FIRRE	EIF2AK3	0.408316782	1.36E-23
FIRRE	EIF2AK2	0.408328314	1.36E-23
FIRRE	CHMP2B	0.335022602	6.05E-16
FIRRE	BIRC6	0.626085477	2.04E-61
FIRRE	ATG2B	0.522143589	6.22E-40
RAB11B-AS1	VAMP7	-0.350040404	2.35E-17
RAB11B-AS1	USP10	-0.324970779	4.83E-15
RAB11B-AS1	SIRT1	-0.358295007	3.65E-18
RAB11B-AS1	RAB7A	-0.321930194	8.91E-15
RAB11B-AS1	PIK3R4	-0.424176953	1.62E-25
RAB11B-AS1	PEX3	-0.352755027	1.28E-17
RAB11B-AS1	NFKB1	-0.371001989	1.87E-19
RAB11B-AS1	NFE2L2	-0.367478845	4.32E-19
RAB11B-AS1	NCKAP1	-0.386736941	3.90E-21
RAB11B-AS1	NAF1	-0.339333786	2.42E-16
RAB11B-AS1	MTOR	-0.319280676	1.51E-14
RAB11B-AS1	MBTPS2	-0.382826961	1.04E-20
RAB11B-AS1	MAPK1	-0.38722407	3.45E-21
RAB11B-AS1	MAP2K7	0.30129363	4.78E-13
RAB11B-AS1	MAP1LC3A	0.377692003	3.70E-20
RAB11B-AS1	ITGB1	-0.419554687	6.04E-25
RAB11B-AS1	HSPA8	-0.336012079	4.91E-16
RAB11B-AS1	GNAI3	-0.415554018	1.86E-24
RAB11B-AS1	FOXO3	-0.302776485	3.63E-13
RAB11B-AS1	EIF4G1	-0.353547373	1.07E-17
RAB11B-AS1	EIF2S1	-0.322388095	8.13E-15
RAB11B-AS1	EIF2AK3	-0.304404232	2.67E-13
RAB11B-AS1	EIF2AK2	-0.338353032	2.99E-16
RAB11B-AS1	EDEM1	-0.364453974	8.79E-19
RAB11B-AS1	BIRC6	-0.305110956	2.34E-13
RAB11B-AS1	BECN1	-0.31318785	5.00E-14
RAB11B-AS1	ATG16L2	0.324723157	5.08E-15
RAB11B-AS1	ATG16L1	-0.301721876	4.41E-13
RAB11B-AS1	ATF6	-0.306838338	1.69E-13
RAB11B-AS1	ARNT	-0.322264444	8.34E-15
AC003102.1	ULK3	0.355676499	6.63E-18
AC003102.1	ATG16L2	0.375825357	5.83E-20

### Prognosis evaluation of the autophagy-related lncRNA signature in patients with EC in the training data set

**Figure 2:**
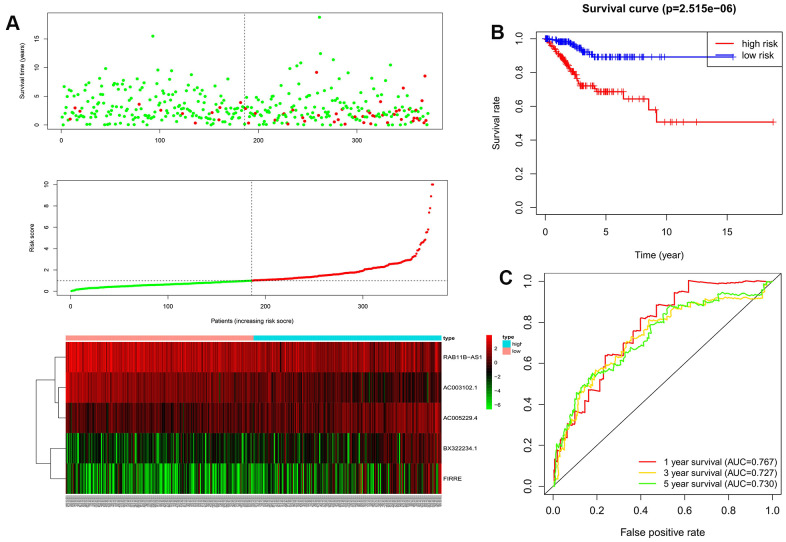
**The evaluation of the autophagy-related lncRNA signature in the training dataset.** (**A**) Autophagy-related lncRNA risk score analysis (Risk score distribution of the EC patients; survival status and duration of the EC patients; Heatmap of the 5 lncRNAs expression). (**B**) Kaplan-Meier survival analysis for EC patients in the training dataset; (**C**) Time-dependent ROC curve analysis for EC patients in the training dataset.

We used the above formula to calculate the prognosis risk score for each patient in the training data set. The patients were divided into high- and low-risk groups by using the median score as the cutoff. The distributions of the risk scores, survival status, and survival duration of the 372 EC patients and the expression heatmap for the 5 lncRNAs are shown in Figure 2A. The K-M survival curve showed that OS was significantly worse for EC patients in the high-risk group than for those in the low-risk group (*P*<0.001, Figure 2B). ROC curves of the 1-, 3-, and 5-year OS rates drawn to evaluate the sensitivity and specificity of the prognosis signature revealed AUCs of 0.767, 0.727, and 0.730, respectively (Figure 2C). This indicates that the prognosis signature could be used to predict the prognosis of EC patients in the training data set.

### Validation of the autophagy-related lncRNA signature in the testing and entire data sets

**Figure 3:**
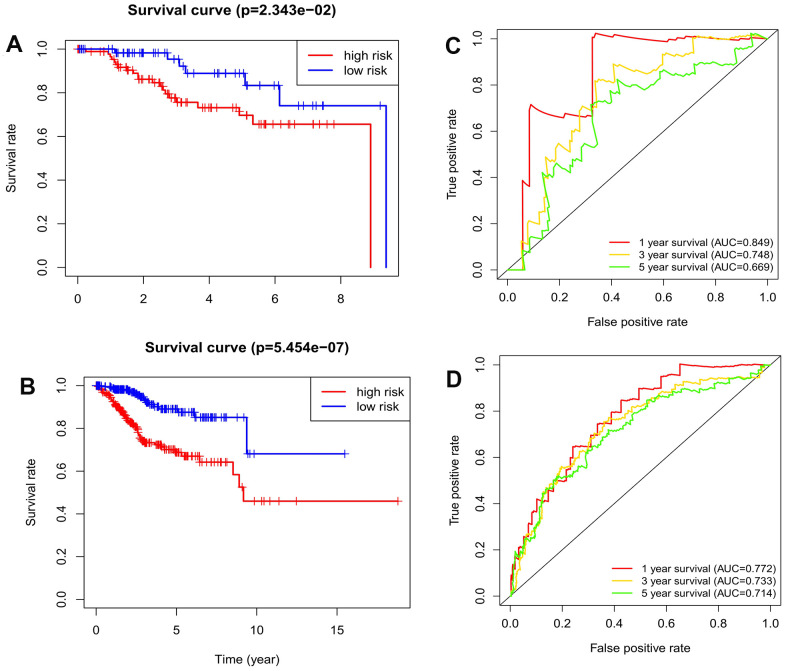
**The validation of the autophagy-related lncRNA signature in the testing dataset and entire dataset.** (**A**) Kaplan-Meier survival analysis for EC patients in the testing dataset; (**B**) Kaplan-Meier survival analysis for EC patients in the entire dataset; (**C**) Time-dependent ROC curve analysis for EC patients in the testing dataset. (**D**) Time-dependent ROC curve analysis for EC patients in the entire dataset.

We also tested the predictive power of the prognosis signature in the testing data set (*n*=156) and the entire data set (*n*=528). The formula was used to calculate the risk scores for EC patients in the testing data set and in the entire data set, and then the EC patients were divided into high- and low-risk groups using the cutoff for the training data set. K-M survival curves for the testing data set and the entire data set showed that the OS remained lower for EC patients in the high-risk group than for those in the low-risk group (Figure 3A, 3B). The AUCs for 1-, 3-, and 5-year OS rates were 0.849, 0.748, and 0.669, respectively, in the testing data set, and 0.772, 0.733, and 0.714 in the entire data set (Figure 3C, 3D). This reverification process showed that the prognosis signature had good accuracy and robustness.

### Independence of the autophagy-related lncRNA signature for EC patients

**Figure 4:**
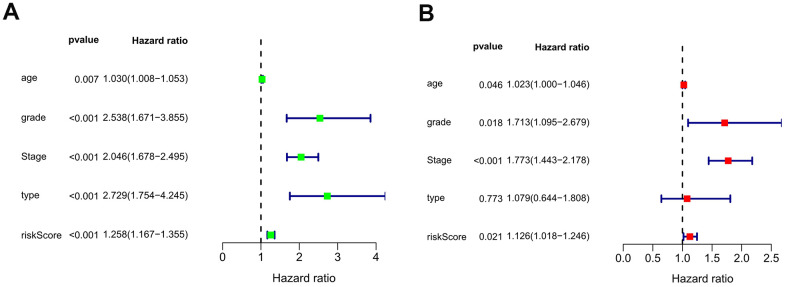
****The forest plots of univariate (**A**) and multivariate (**B**) Cox regression analysis of the prognostic value in the entire dataset.

The independent value of the autophagy-related lncRNA prognosis signature was evaluated by performing univariate and multivariate Cox regression analyses of the model and the clinical prognostic factors in the entire data set. The clinical prognostic factors comprised age, pathological type (endometrioid adenocarcinoma versus mixed and serous adenocarcinoma), FIGO stage (stage I + stage II versus stage III + stage IV), and pathological grade (grade 1 + grade 2 versus grade 3). The univariate Cox regression analysis showed that the autophagy-related lncRNA prognosis signature and the pathological type, age, FIGO stage, and tumor pathological grade were associated with the prognosis of EC patients (*P*<0.05) (Figure 4A). Meanwhile, the multivariate Cox regression analysis showed that the autophagy-related lncRNA prognosis signature and age, FIGO stage, and tumor pathological grade were independent prognostic factors for EC patients, whereas the pathological type was not (Figure 4B).

**Figure 5:**
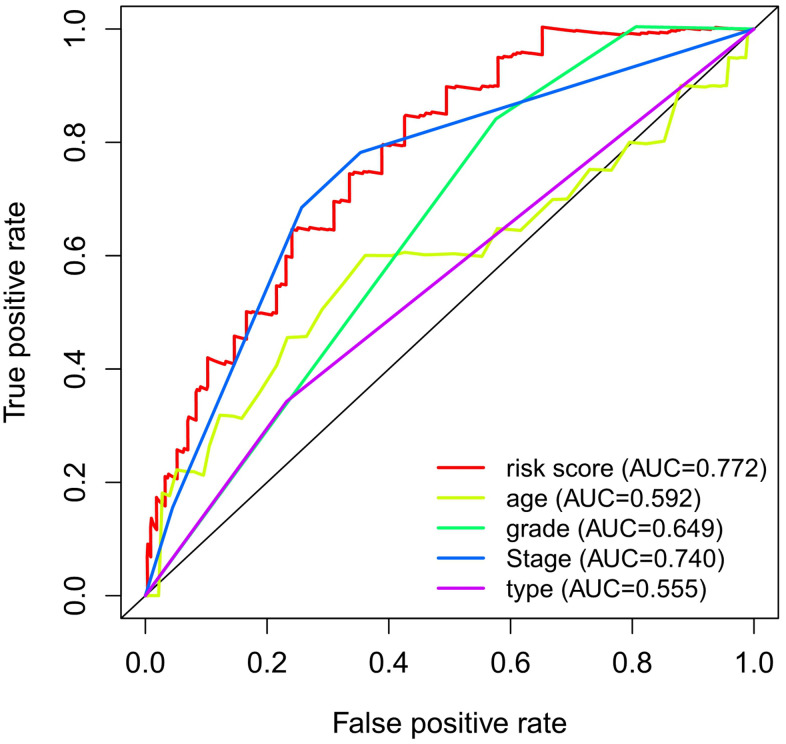
**ROC curve analysis for 1-year OS in the entire dataset.**

The prognostic effects of the autophagy-related gene prognosis signature were compared with those of other clinical factors by drawing ROC curves for the 1-year OS. The AUC was 0.772 for the autophagy-related lncRNA prognosis signature, and 0.555, 0.592, 0.740, and 0.649 for the pathological type, age, FIGO stage, and pathological grade, respectively. These values indicate that our autophagy-related lncRNA prognosis signature has better prognostic potential than the other clinical factors (Figure 5).

### Clinical utility of the autophagy-related lncRNA signature

**Figure 6:**
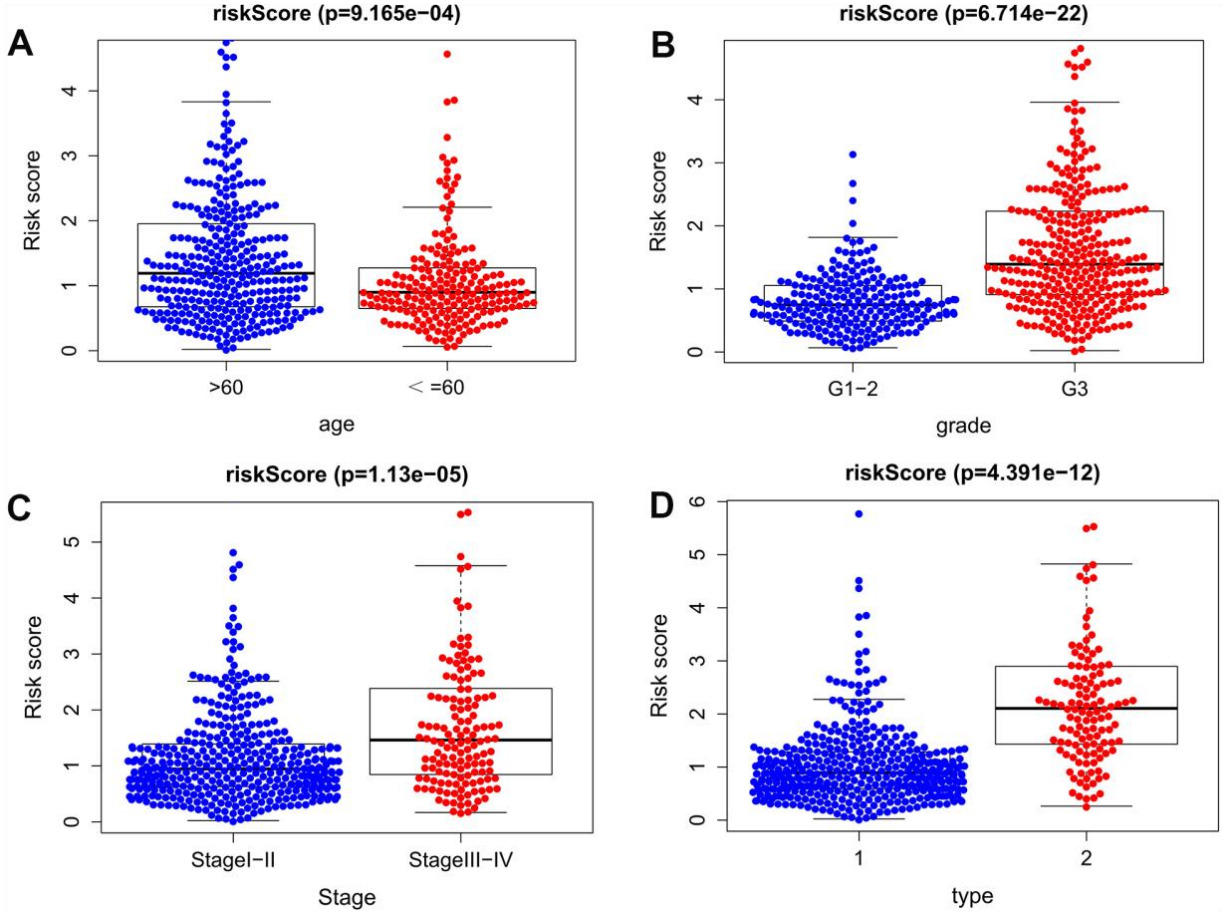
**Clinical significance of the prognostic signature of EC.** (**A**) age; (**B**) pathological grade; (**C**) FIGO stage; (**D**) histological type (1 endometrioid adenocarcinoma, 2 mixed and serous adenocarcinoma).

We further analyzed the relationships between the autophagy-related lncRNA prognosis signature and age, pathological grade, FIGO grade, and pathological type of EC patients. The results show that, the difference of the risk score for our signature was observed between age > 60 and age ≤ 60 (*P* <0.001). Besides, the risk score for our signature was higher in Stage III-IV than in Stage I-II (*P* <0.001), and higher in G3 than G1-2 (*P* <0.001), and higher in mixed and serous adenocarcinoma than endometrioid adenocarcinoma(P < 0.001) (Figure 6). The above results fully prove that the signature is closely related to EC progression.

### Gene set enrichment analysis

**Figure 7:**
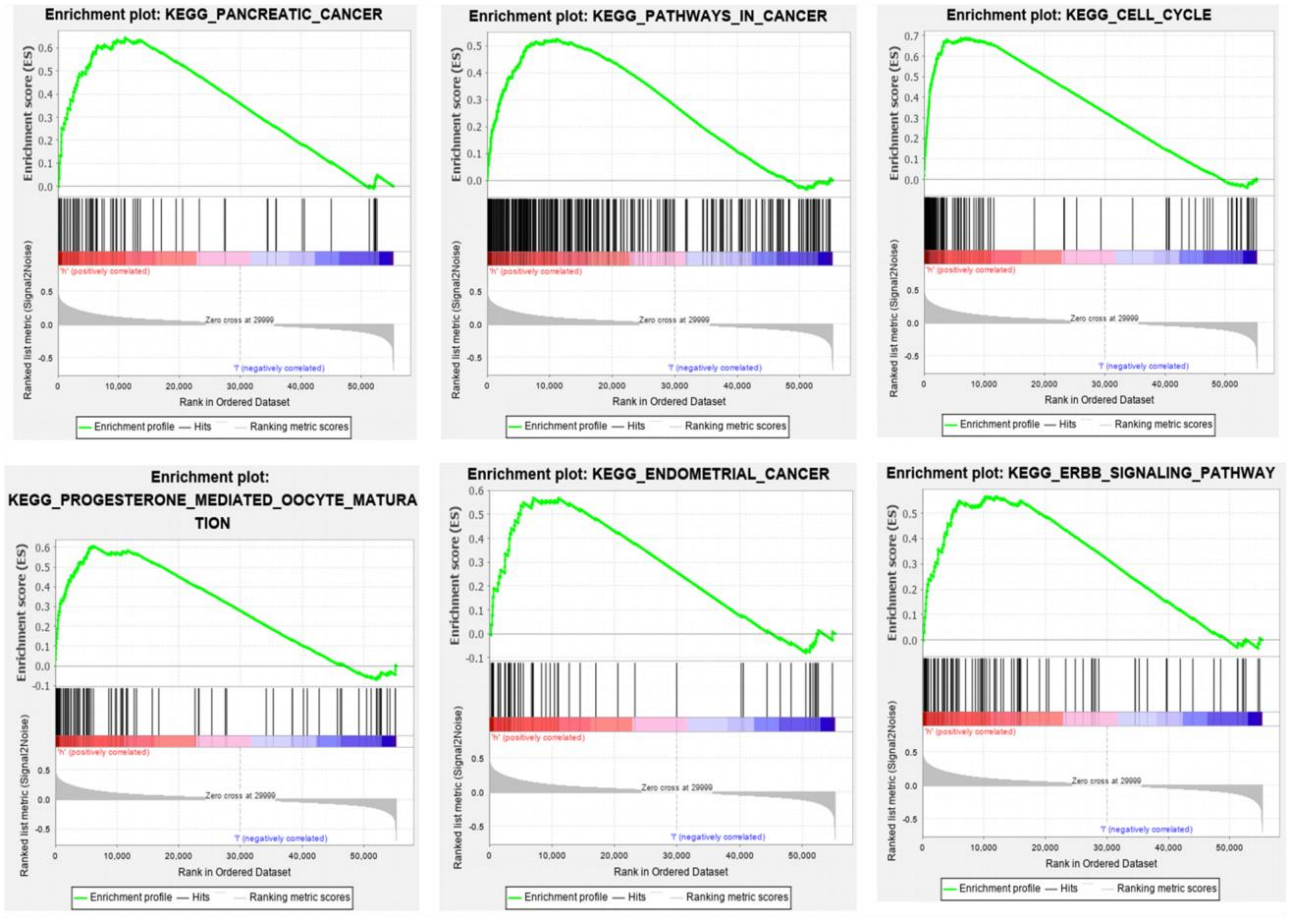
**Some pathways were enriched in the high-risk group, among which the landmark cancer-related pathways were enriched.**

**Figure 8:**
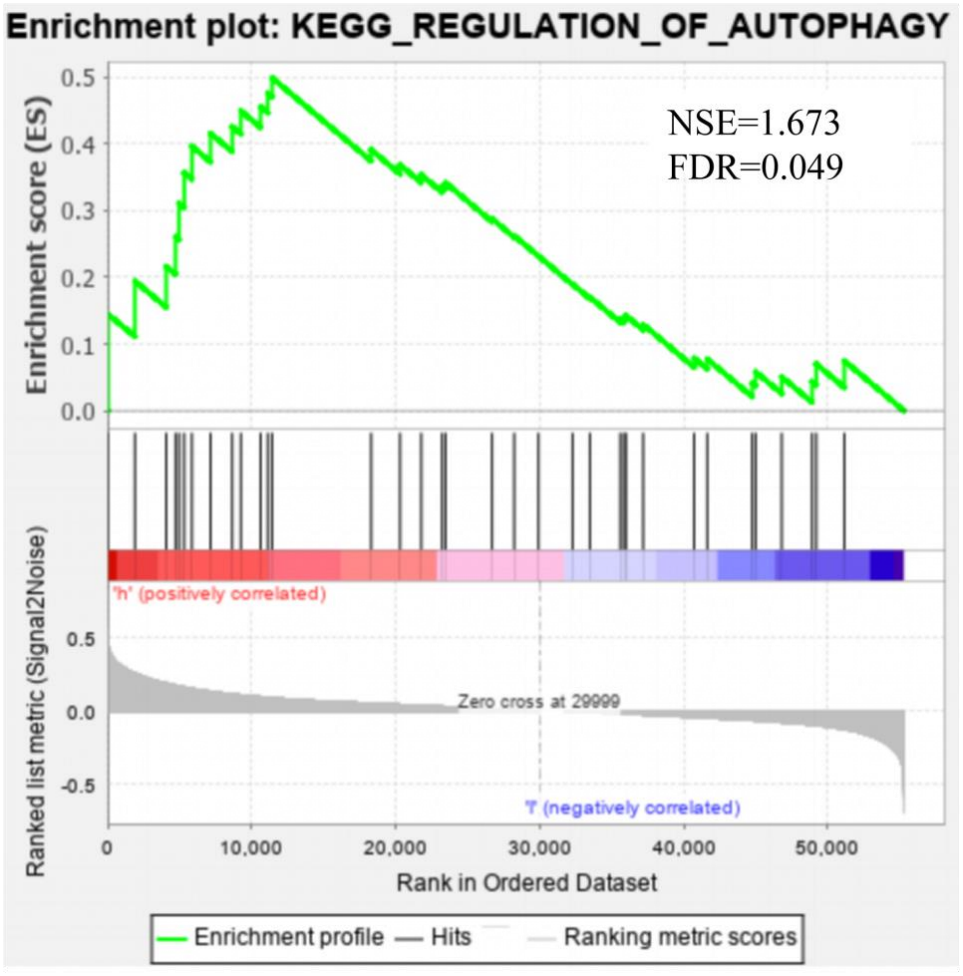
**Gene set enrichment analysis showed that the autophagy pathway was enriched in the high-risk group.**

GSEA was applied to the high- and low-risk groups of the autophagy-related lncRNA prognosis signature. The results revealed that 69 pathways were significantly enriched in the high-risk group, including those related to axon guidance, progesterone-mediated oocyte maturation, cancer, ErbB signaling, DNA replication, EC, MAPK, and the cell cycle (false discovery rate: *q*<0.05) (Table 3). Figure 7 shows that there was partial pathway enrichment in the high-risk group, including in landmark-cancer-related pathways. We similarly found that autophagy-related signaling pathways were also enriched in the high-risk group (Figure 8), further confirming that the identified autophagy-related lncRNAs contribute to important cancer and autophagy pathways, which might represent strong evidence for its usefulness in the development of targeted therapies for EC.

**Table 3:** Results of gene set enrichment analysis based on the autophagy-related lncRNA signature.

**Name**	**Size**	**ES**	**NES**	**NOM *p*-val**	**FDR *q*-val**	**FWER *p*-val**
KEGG_AXON_GUIDANCE	129	0.609	2.315	0.000	0.002	0.002
KEGG_CELL_CYCLE	124	0.689	2.223	0.002	0.004	0.010
KEGG_PROGESTERONE_MEDIATED_OOCYTE_MATURATION	85	0.606	2.201	0.000	0.005	0.013
KEGG_PANCREATIC_CANCER	70	0.644	2.229	0.000	0.006	0.009
KEGG_CHRONIC_MYELOID_LEUKEMIA	73	0.621	2.142	0.000	0.006	0.023
KEGG_OOCYTE_MEIOSIS	112	0.594	2.108	0.004	0.007	0.032
KEGG_ERBB_SIGNALING_PATHWAY	87	0.566	2.115	0.000	0.007	0.031
KEGG_PATHWAYS_IN_CANCER	325	0.525	2.146	0.000	0.007	0.023
KEGG_SMALL_CELL_LUNG_CANCER	84	0.592	2.124	0.000	0.008	0.029
KEGG_UBIQUITIN_MEDIATED_PROTEOLYSIS	134	0.588	2.077	0.004	0.008	0.039
KEGG_NEUROTROPHIN_SIGNALING_PATHWAY	126	0.548	2.072	0.002	0.008	0.044
KEGG_ADHERENS_JUNCTION	73	0.614	2.082	0.000	0.008	0.038
KEGG_ENDOCYTOSIS	181	0.508	2.058	0.000	0.008	0.051
KEGG_NON_SMALL_CELL_LUNG_CANCER	54	0.617	2.153	0.000	0.009	0.023
KEGG_GLIOMA	65	0.554	2.014	0.000	0.009	0.075
KEGG_TIGHT_JUNCTION	132	0.500	2.016	0.002	0.009	0.072
KEGG_MAPK_SIGNALING_PATHWAY	267	0.481	2.025	0.000	0.009	0.067
KEGG_REGULATION_OF_ACTIN_CYTOSKELETON	213	0.518	2.047	0.000	0.009	0.055
KEGG_BASAL_TRANSCRIPTION_FACTORS	35	0.675	2.026	0.002	0.009	0.066
KEGG_COLORECTAL_CANCER	62	0.585	2.016	0.002	0.009	0.072
KEGG_MISMATCH_REPAIR	23	0.804	2.001	0.002	0.009	0.085
KEGG_INSULIN_SIGNALING_PATHWAY	137	0.502	2.026	0.002	0.009	0.065
KEGG_RNA_DEGRADATION	59	0.654	2.031	0.002	0.009	0.063
KEGG_RENAL_CELL_CARCINOMA	70	0.576	2.037	0.000	0.009	0.06
KEGG_INOSITOL_PHOSPHATE_METABOLISM	54	0.583	1.978	0.002	0.012	0.102
KEGG_GAP_JUNCTION	90	0.523	1.963	0.002	0.013	0.116
KEGG_SPLICEOSOME	127	0.647	1.963	0.014	0.013	0.115
KEGG_ONE_CARBON_POOL_BY_FOLATE	17	0.749	1.939	0.004	0.015	0.146
KEGG_FOCAL_ADHESION	199	0.516	1.943	0.008	0.015	0.14
KEGG_DNA_REPLICATION	36	0.819	1.948	0.004	0.015	0.137
KEGG_TGF_BETA_SIGNALING_PATHWAY	85	0.542	1.910	0.008	0.017	0.176
KEGG_B_CELL_RECEPTOR_SIGNALING_PATHWAY	75	0.560	1.914	0.012	0.017	0.172
KEGG_PURINE_METABOLISM	157	0.478	1.919	0.002	0.017	0.166
KEGG_ENDOMETRIAL_CANCER	52	0.569	1.914	0.002	0.018	0.171
KEGG_TYPE_II_DIABETES_MELLITUS	47	0.556	1.897	0.002	0.018	0.186
KEGG_PROSTATE_CANCER	89	0.521	1.920	0.000	0.018	0.165
KEGG_FC_GAMMA_R_MEDIATED_PHAGOCYTOSIS	96	0.531	1.900	0.008	0.018	0.183
KEGG_WNT_SIGNALING_PATHWAY	150	0.496	1.902	0.000	0.018	0.179
KEGG_PYRIMIDINE_METABOLISM	98	0.538	1.872	0.008	0.021	0.214
KEGG_ARRHYTHMOGENIC_RIGHT_VENTRICULAR_CARDIOMYOPATHY_ARVC	74	0.516	1.866	0.004	0.021	0.218
KEGG_THYROID_CANCER	29	0.587	1.862	0.010	0.021	0.221
KEGG_RNA_POLYMERASE	29	0.644	1.838	0.015	0.024	0.25
KEGG_PATHOGENIC_ESCHERICHIA_COLI_INFECTION	56	0.557	1.840	0.012	0.024	0.249
KEGG_T_CELL_RECEPTOR_SIGNALING_PATHWAY	108	0.529	1.834	0.004	0.024	0.256
KEGG_HOMOLOGOUS_RECOMBINATION	28	0.696	1.841	0.022	0.024	0.247
KEGG_DILATED_CARDIOMYOPATHY	90	0.498	1.844	0.006	0.024	0.245
KEGG_LYSINE_DEGRADATION	44	0.567	1.812	0.027	0.027	0.287
KEGG_DORSO_VENTRAL_AXIS_FORMATION	24	0.610	1.813	0.008	0.028	0.283
KEGG_MELANOGENESIS	101	0.471	1.805	0.008	0.028	0.297
KEGG_ACUTE_MYELOID_LEUKEMIA	57	0.518	1.799	0.016	0.029	0.299
KEGG_ECM_RECEPTOR_INTERACTION	84	0.541	1.777	0.016	0.031	0.324
KEGG_ADIPOCYTOKINE_SIGNALING_PATHWAY	67	0.486	1.779	0.010	0.032	0.322
KEGG_MTOR_SIGNALING_PATHWAY	52	0.482	1.781	0.020	0.032	0.32
KEGG_PHOSPHATIDYLINOSITOL_SIGNALING_SYSTEM	76	0.496	1.766	0.006	0.033	0.337
KEGG_NOTCH_SIGNALING_PATHWAY	47	0.519	1.755	0.012	0.034	0.352
KEGG_BASAL_CELL_CARCINOMA	55	0.537	1.755	0.012	0.034	0.352
KEGG_TOLL_LIKE_RECEPTOR_SIGNALING_PATHWAY	102	0.491	1.757	0.028	0.035	0.351
KEGG_LONG_TERM_POTENTIATION	70	0.476	1.747	0.014	0.035	0.364
KEGG_BLADDER_CANCER	42	0.494	1.729	0.016	0.039	0.387
KEGG_PROXIMAL_TUBULE_BICARBONATE_RECLAMATION	23	0.575	1.725	0.012	0.040	0.393
KEGG_CYTOSOLIC_DNA_SENSING_PATHWAY	54	0.503	1.722	0.028	0.040	0.401
KEGG_NUCLEOTIDE_EXCISION_REPAIR	44	0.607	1.710	0.035	0.043	0.424
KEGG_ALANINE_ASPARTATE_AND_GLUTAMATE_METABOLISM	30	0.500	1.696	0.010	0.045	0.444
KEGG_MELANOMA	71	0.449	1.697	0.015	0.046	0.439
KEGG_PYRUVATE_METABOLISM	40	0.516	1.685	0.029	0.046	0.466
KEGG_SELENOAMINO_ACID_METABOLISM	25	0.539	1.688	0.031	0.047	0.466
KEGG_JAK_STAT_SIGNALING_PATHWAY	155	0.428	1.685	0.026	0.047	0.466
KEGG_REGULATION_OF_AUTOPHAGY	35	0.499	1.673	0.026	0.049	0.489
KEGG_HYPERTROPHIC_CARDIOMYOPATHY_HCM	83	0.451	1.675	0.015	0.049	0.486

* SIZE indicates the number of genes in the gene set; ES represents enrichment score; NES represents normalized enrichment score; NOM *p*-val represents nominal *p* value; FDRq-val represents false discovery rate; FWERp-val is Family-wise error rate.

## DISCUSSION

lncRNA has been shown to play an important role in the development and progression of tumors, including EC [36], and can be used as a biomarker for the diagnosis, prognosis, and potential therapeutic targets in various cancers. Recent studies of lncRNAs have identified that many are involved in the regulation of autophagy in tumors, and that most autophagy-related lncRNAs affect the occurrence and development of tumors [37]. Therefore, autophagy-related lncRNAs are a potential and promising target for tumor treatments and prognosis evaluations. Zhou et al. developed a signature based on 13 autophagy-related lncRNAs that could serve as an independent prognosis indicator for lung adenocarcinoma [38], and Luan et al. identified 10 prognostic autophagy-related lncRNAs and validated an autophagy-related-lncRNA-based index for predicting the OS in glioma [39]. However, the prognostic significance of autophagy-related lncRNAs in EC has not been reported previously.

The present study collected expression data of lncRNAs and autophagy-related genes of EC patients in the TCGA database, and evaluated the correlations between lncRNAs and autophagy-related genes using Pearson correlation analysis in order to identify autophagy-related lncRNAs. The obtained samples were randomly divided into training and testing data sets at the proportion of 7:3. In the training data set, we constructed a novel autophagy-related lncRNA prognosis signature using univariate and multivariate Cox regression analyses. After dividing the EC patients into high- and low-risk groups, those in the high-risk group had a worse OS. In addition, our signature was found to be a more-effective independent prognostic factor for EC compared with traditional clinical prognostic factors, and have a good AUC (i.e., higher prognosis resolution). This study also analyzed the relationships between the autophagy-related lncRNA prognosis signature and clinical features, with the results showing that the risk score for the signature tended to increase at higher levels, suggesting that the signature reflects the progression of EC.

Our signature indicates that EC patients with high expression levels of AC005229.4, BX322234.1, and FIRRE have worse survival, while those with high expression levels of RAB11B-AS1 and AC003102.1 have better survival. RAB11B-AS1 can inhibit the development of osteosarcoma via its natural antisense transcript RAB11B, and its low expression level is associated with a poor prognosis of osteosarcoma patients [40]. Shi et al. found that FIRRE lncRNA was overexpressed in diffuse large-B-cell lymphoma (DLBCL) tissue and cells. FIRRE lncRNA can promote the proliferation of tumor cells, reduce cell apoptosis, and is associated with poor OS in DLBCL patients [41]. However, there have been no previous reports on the other three lncRNAs identified in the present study: AC005229.4, BX322234.1, and AC003102.1.

Our GSEA also showed that cancer-related pathways were significantly enriched in the high-risk group, including those related to pancreatic cancer, small-cell lung cancer, EC, cancer, ErbB signaling, MAPK, and other common cancers [42, 43]. Moreover, the autophagy-related signaling pathways were also enriched in the high-risk group. This suggests that the five autophagy-related lncRNAs that we have identified are related to the occurrence and development of EC.

This study was subject to some limitations. First, all of the analyzed data were collected from the TCGA database, and so our novel signature needs to be further validated in other prospective cohorts in order to ensure its robustness. Second, the potential and molecular correlations between our autophagy-related lncRNAs and autophagy need to be studied further. Third, the role and mechanism of these autophagy-related lncRNAs in EC also need to be further validated.

In summary, we have constructed an autophagy–lncRNA coexpression network to explore the molecular markers related to the progression and prognosis of EC, and have developed a signature based on five autophagy-related lncRNAs that has independent prognostic value for EC patients.

## MATERIALS AND METHODS

### Collection of data on EC patients

The transcriptome profiling data of EC and corresponding clinical information were extracted from the TCGA database at https://portal.gdc.cancer.gov/. The EC data set totaled 552 tumor samples, with clinical follow-up data being available for 528 of the samples. We randomly divided EC patients with clinical follow-up data at the proportion of 7:3 into a training data set (*n*=372) and a testing data set (*n*=156). The training data set was used to identify autophagy-related lncRNAs related to the prognosis of EC and to establish a prognosis signature, whose validity and stability were verified in the testing data set (Table 4).

**Table 4:** Clinical characteristics of EC patients from each database.

**Characteristics**	**Training dataset (n=372)**		**Testing dataset (n=156)**		**Entire dataset (n=528)**		***P*-value**
	**n**	**%**	**n**	**%**	**n**	**%**	
Age (year)							0.902
≤60	140	37.63%	62	39.74%	202	38.26%	
>60	232	62.37%	94	60.26%	326	61.74%	
FIGO stage							
I	234	62.90%	98	62.82%	332	62.88%	0.967
II	33	8.87%	18	11.54%	51	9.66%	
III	85	22.85%	34	21.79%	119	22.54%	
IV	20	5.38%	6	3.85%	26	4.92%	
Histological type							0.194
Endometrioid	292	78.49%	111	71.15%	403	76.33%	
Mixed and serous	80	21.51%	45	28.85%	125	23.67%	
Tumor grade							0.198
G1	73	19.62%	25	16.02%	98	18.56%	
G2	93	25.00%	27	17.31%	120	22.73%	
G3	206	55.38%	104	66.67%	310	58.71%	

### Identification of autophagy-related lncRNA

The lncRNA data and autophagy-related genes were extracted from the transcriptome profiling data of EC obtained from the TCGA database. The list of autophagy genes was obtained from the Human Autophagy Database at http://autophagy.lu/clustering/index.html. Pearson correlation analysis was used to calculate the correlations between lncRNAs and autophagy-related genes. Any lncRNA with a correlation coefficient of >0.3 and *P*<0.001 was regarded as being related to autophagy.

### Construction of a prognosis signature based on autophagy-related lncRNAs

Univariate Cox regression analyses were applied to the training data set to evaluate the prognostic value of autophagy-related lncRNAs. lncRNAs for which *P*<0.01 were then analyzed by stepwise multivariate Cox regression. According to the principle of the minimum Akaike information criterion, a prognosis signature based on autophagy-related lncRNA was constructed using the following formula: $\text{PI}=\sum_{i=1}^{n}\left(\beta \text{i}*{\text{lncRNA}}_{\text{i}}\right),$ where *β_i_* and [lncRNA_*i*_] are the regression coefficient and expression value of the *i*-th autophagy-related lncRNA, respectively, and *n* is the number of autophagy-related lncRNAs included in the prognosis signature. This formula was used to calculate the risk score for each EC patient, and then all of the EC patients were divided into high- and low-risk groups using the median risk score as the cutoff. Kaplan-Meier (K-M) survival analysis was then used to compare the OS rate between the high- and low-risk groups, with a log-rank *P* of <0.05 for the survival difference between the two groups considered to be statistically significant.

The receiver operating characteristics (ROC) curve and the area under the ROC (AUC) were used to evaluate the sensitivity and specificity of the autophagy-related lncRNA prognosis signature. We also analyzed the relationship between this signature and other clinical factors related to the prognosis of EC, and further compared the survival prediction capabilities of the prognostic factors.

### Gene set enrichment analysis

Gene set enrichment analysis (GSEA) was applied to the high- and low-risk groups of the autophagy-related lncRNA prognosis signature. This study verified whether the genes that were differentially expressed between the two groups are enriched during autophagy. In addition, we analyzed whether the autophagy pathway was enriched in the GSEA high-risk group.

### Statistical analysis

Statistical analyses were implemented using R software (version 3.6.2). Pearson correlation analysis was used to evaluate the correlations between autophagy genes and lncRNA. Survival analysis was performed by the K-M method, with the log-rank test used for comparisons. The ROC curve analysis was performed using the survivalROC package, while Cytoscape software (version 3.71) was used to construct an autophagy–lncRNA coexpression network. The Gene Set Enrichment Analysis software (version 4.0.3) was used for the GSEA.
